# Effective mitigation strategy in early stage of COVID-19 pandemic in China

**DOI:** 10.1186/s40249-020-00759-3

**Published:** 2020-10-12

**Authors:** Xiao-Yue Yu, Chen Xu, Hu-Wen Wang, Rui-Jie Chang, Yin-Qiao Dong, Lhakpa Tsamlag, Shu-Xian Zhang, Yue-Lin Yu, Ru-Si Long, Hui Wang, Yong Cai

**Affiliations:** 1 School of Public Health, Shanghai Jiao Tong University School of Medicine, Shanghai, China; 2 Department of Environmental and Occupational Health, School of Public Health, China Medical University, Shenyang, China

**Keywords:** COVID-19, Public health control measure

## Abstract

In the past five months, success in control the national epidemic of coronavirus disease 2019 (COVID-19) has been witnessed in China. The implementation of public health measures accounts for the success which include different interventions in the early or later stages of the outbreak. It is clear that although not all measures were universally effective worldwide, their achievements have been significant. More solidarity is needed to deal with this global pandemic with more learning and understanding. Understanding which of the public health interventions implemented in China were effective may provide ideas for international epidemic control.

## Background

**Fig. 1:**
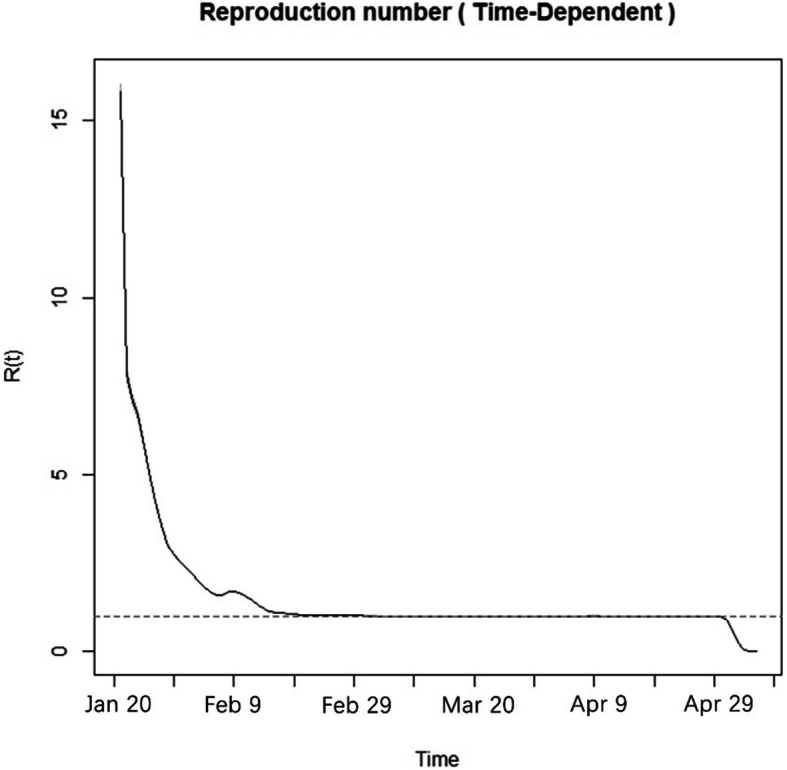
Estimates of the effective reproductive number in China over time. (Data were obtained from the website of the National Health Commission of the People’s Republic of China)

Aggressive COVID-19 epidemic has hit the health system around the world. In China, a peak-and-fall trend in the national epidemic of COVID-19 was witnessed. We performed an SEIR model (Susceptible, Exposed, Infectious and Removed model) for analysing epidemic trends in China shown in Fig. 1, which indicates the outbreak in China has been under control since mid-February.

According to data from the National Health Commission, People’s Republic of China, the number of infected individuals in Wuhan was about 50 000 at the end of February, no inflection points or peaks in cases were expected at the same time [1]. The epidemic in Wuhan was expected to continue to expand over time if effective prevention and control measures were not consistently implemented [2]. At present, community transmission has been controlled nationwide. The implementation of public health measures, such as reduction of the flow of people and centralized isolation during the early stages of the outbreak as well as population symptom screening in the later stages, accounts for the success in controlling the explosive growth of the COVID-19 epidemic in China within two months. The effectiveness of public health interventions in breaking the chain of transmission has been demonstrated by multiple lines of evidence. Although, it is clear that not all measures were universally effective worldwide, their achievements have been significant. COVID-19 has become a true pandemic, it is imperative for the world to forge a consensus on epidemic control. Understanding which of the public health interventions implemented in China were effective may provide ideas for international pandemic control.

## Reduction of the flow of people is the key mitigation measure

Lessons learnt from the influenza pandemic of the early twentieth century in the United States showed that the timing of interventions was crucial for pandemic control: cities with rapid responses had lower excess death rates than cities with slower responses [3]. Hence it is effective to be prepared and actively respond as soon as possible.

The outbreak of COVID-19 in Wuhan, China coincided with Chinese New Year, and the massive flow of people during the early stages of the Spring Festival promoted the spread of the epidemic. Although there was no effective control of the flow of people at the beginning of the epidemic in the epicentre, a series of measures such as the closure of Wuhan city, traffic control and closed management in community was followed, which has effectively controlled the epidemic. Recent studies found that the closure of Wuhan’s outbound routes postponed outbreaks in other cities by 2.91 days, stopping further spread of the virus and buying time for other cities across the country to prepare for their own epidemics. Among public health interventions, traveling restrictions, quarantine and isolation are the most effective in the early stage of outbreak in Wuhan [4–6].

While Wuhan has implemented various measures to restrict the flow of people, health authorities encouraged people across the country to “stay at home” to avoid unnecessary gatherings and public activities, extended the Spring Festival holiday, and postponed the start of the spring semester. Communities worked with health authorities were responsible for resident information management, outbreak-related education and symptom investigation. In general, the role of “social distancing” and preventive public health policies for epidemic containment were widely understood by the public.

## “Early detection and early isolation” coupled with medical services are important interventions

Traditional public health interventions undoubtedly played a major role in the containment of the epidemic because no drugs or vaccines have yet been developed against the virus [6]. The detection time and procedures have also been continuously shortened and optimised, which benefits large-scale population screening and case diagnosis. Early detection combined with expansion of the scope of screening worked well as a strategy not only in Wuhan, China, but also in Republic of Korea. Many novel rapid detection methods such as drive-through and walking-through detection station were applied in Republic of Korea, enabled the control of community transmission in a short period of time without closure of industries and schools. Rapid testing enables early treatment and full use of existing primary medical institutions, especially hospitals that were specifically designed for centralised isolation, diagnosis, and treatment, and hospitals offered tiered medical services according to the severity of disease. Furthermore, isolating confirmed cases in a family has proved to be counterproductive for epidemic control in China, leading to more infections. So centralised isolation is required to prevent transmission in families.

During this process, priority always was given to epidemic control. There is no doubt that authorities must reach a consensus with the public, as many limitations and restrictions require a high level of participation from the entire community.

## Strengthen population screening is a long-term approach in fighting COVID-19 pandemic

Since the COVID-19 outbreak is going to last for a long time worldwide, mass screening which already proved successful in Republic of Korea and disinfection in public places is needed to avoid community communication especially in high risks areas. Containment measures proved to be effective in the early stages of the epidemic also need to be taken seriously in the future, including increasing the production of nucleic acid detection reagents, adopting tailored prevention and control strategies based on different epidemic situations, ensuring sufficient centralising medical resources and encouraging social distancing. Once community communication stars again, second outbreak wave could hardly be prevented. While, we need to constantly monitor reproduction number of COVID-19 and adjust the extent of limitation policies according to the epidemic situation. At the same time, it is also important to solve social contradiction and conflicts caused by limitation policies. Broad solidarity of social groups is required. Policy support for vulnerable populations and industries should be strengthened, especially those below the poverty line and tertiary industries, as COVID- 19 epidemic worsen poverty and inequality by increasing unemployment.

## Conclusions

Although different countries and regions are experiencing different epidemic situations of COVID-19, the mitigation and suppression measures are effective from China’s experience, which needed to be continuously implemented. According to the severity of the epidemic situation, the extent of public health policies can be adjusted appropriately. Whereas support for vulnerable population and industries is equally important.

## Data Availability

All data were obtained from the website of the National Health Commission of the People’s Republic of China with open access.

## Abbreviations

SEIR model: Susceptible, exposed, infectious and removed model
COVID-19: Coronavirus disease 2019
